# When does prior knowledge disproportionately benefit older adults’ memory?

**DOI:** 10.1080/13825585.2015.1099607

**Published:** 2015-10-16

**Authors:** Stephen P. Badham, Mhairi Hay, Natasha Foxon, Kiran Kaur, Elizabeth A. Maylor

**Affiliations:** ^a^ Department of Psychology, University of Warwick, Coventry, UK

**Keywords:** Aging, prior knowledge, free recall, cued recall, recognition, memory

## Abstract

Material consistent with knowledge/experience is generally more memorable than material inconsistent with knowledge/experience – an effect that can be more extreme in older adults. Four experiments investigated knowledge effects on memory with young and older adults. Memory for familiar and unfamiliar proverbs (Experiment 1) and for common and uncommon scenes (Experiment 2) showed similar knowledge effects across age groups. Memory for person-consistent and person-neutral actions (Experiment 3) showed a greater benefit of prior knowledge in older adults. For cued recall of related and unrelated word pairs (Experiment 4), older adults benefited more from prior knowledge only when it provided uniquely useful additional information beyond the episodic association itself. The current data and literature suggest that prior knowledge has the age-dissociable mnemonic properties of (1) improving memory for the episodes themselves (age invariant), and (2) providing conceptual information about the tasks/stimuli extrinsically to the actual episodic memory (particularly aiding older adults).

Personal concerns related to memory decline are evident in healthy older adults (Hertzog & Hultsch, 2000); memory decline is also one of the most salient aspects of cognitive aging experimentally (Zacks, Hasher, & Li, 2000), impacting older adults’ lives socially and culturally (Naveh-Benjamin & Ohta, 2012). Given that older adults have built up an extensive body of knowledge across their lifespan (e.g., Verhaeghen, 2003), and that they have largely intact access to that knowledge (e.g., Laver & Burke, 1993; Umanath & Marsh, 2014), it is crucial to understand how older adults can utilize their knowledge to improve memory.

An individual’s structured knowledge of the world can influence how information is stored and retrieved from memory (Bartlett, 1932). Information consistent with prior knowledge can be more memorable than information inconsistent with prior knowledge across a wide variety of paradigms. For example, Bransford and Johnson (1972) demonstrated that participants were better able to comprehend and remember passages of text when they were able to link the content to their knowledge of the world. Chase and Simon (1973) showed that expert chess players were better able to remember the positions of chess pieces on a chess board than were novice chess players. Other studies have shown that individuals with particular expertise are better than less-knowledgeable individuals at remembering text related to that expertise (e.g., Arbuckle, Vanderleck, Harsany, & Lapidus, 1990; Miller, 2003). Additionally, novel material (material unsupported by existing knowledge) is more difficult to remember than familiar material: words are easier to remember than nonwords and foreign words (e.g., Hulme, Maughan, & Brown, 1991), images of faces are easier to remember than abstract crystal structures and ink blots (Goldstein & Chance, 1970), and possible line drawings of objects are easier to remember than impossible ones (Schacter, Cooper, & Valdiserri, 1992).

Many studies have shown that young and older adults have a differential reliance on knowledge use in memory tasks (see Umanath & Marsh, 2014, for a review). Often, prior knowledge has a larger benefit to memory for older adults relative to young adults. Castel (2005) found that age differences in memory for the prices of items were reduced when those prices were realistic (knowledge-consistent) compared with when they were unrealistic (knowledge-inconsistent). Hess (1985) tested young and older adults’ memory for typical and atypical actions described in scripts. For example, in a script describing a visit to a restaurant, a typical action was looking at the menu and an atypical action was putting a pen in a pocket. Older adults had poorer memory overall, but the age deficit was smaller for typical than for atypical actions. Similarly, for actions in videos, Garcia-Bajos, Migueles, and Aizpurua (2012) found that age and typicality interacted, with significant age deficits in memory for low-typicality actions but not for high-typicality actions. Naveh-Benjamin, Hussain, Guez, and Bar-On (2003) showed that age deficits in memory for word pairs were alleviated by semantic relations between the words compared to unrelated word pairs. Badham, Estes, and Maylor (2012) extended this by showing that this was the case even when the word pairings were novel (e.g., apartment–dog, box–wine) so long as they could be integrated together within a context. Similarly, Smith, Park, Earles, Shaw, and Whitinga (1998) found that age deficits in memory for associations between pictures were alleviated when the pictures were related to one another compared with when they were unrelated to one another.

Umanath and Marsh (2014) argued that access to knowledge is largely preserved in old age and this can differentially support older adults’ episodic memory (relative to young adults’ memory) for information that is consistent with existing knowledge, or differentially hinder older adults’ episodic memory for information that is inconsistent with existing knowledge. Their review presented several theoretical perspectives on these patterns of results in the literature, but crucially did not discuss age invariance in the effects of knowledge on memory tasks, which is a key focus of the current study.

The application of prior knowledge to memory tasks does not always benefit older adults more than young adults and there is sufficient evidence in the literature to conclude that this is not due to Type II errors. For example, Arbuckle et al. (1990) tested young and older music experts and nonmusic experts on their memory for passages of text about music or about dogs. The music experts showed better memory than the nonmusic experts for the music passages, but not for the dog passages. Music expertise benefited young and older adults to the same extent (a result also found with cooking expertise by Miller, 2003). Morrow, Leirer, Carver, and Tanke (1998) assessed memory for appointments in young and older adults where the information was presented in an ordered (knowledge-consistent) or disordered (knowledge-inconsistent) sequence. Memory was improved to the same extent across age groups when the information in the appointments was ordered compared to disordered – a similar result was also found with the ordering of instructions related to taking medication (Morrow, Leirer, Andrassy, Tanke, & Stine-Morrow, 1996). Similarly, Cherry and Jones (1999) tested young and older adults’ memory for the positions of dolls’-house furniture. In a high knowledge consistency condition, the furniture was organized (e.g., all kitchen furniture together), whilst in a low knowledge consistency condition the furniture was unorganized. The positions of organized furniture were remembered better than the positions of unorganized furniture to the same extent in the two age groups. Finally, Gutchess and Park (2009) tested memory for associations between pictures of objects and their backgrounds. Consistent object–background pairings (e.g., a cow in a farm) were remembered better than inconsistent object–background pairings (e.g., a cow in a laundry room), but to the same extent by groups of young and older participants.

## The current study

Overall, the literature presents a mixed set of results with regard to age differences in knowledge use during memory tasks. Some studies show that older adults’ memory is more influenced by prior knowledge than young adults’ memory, whilst other studies show age invariance in this effect. It should be noted that to the present authors’ knowledge there are only two studies where prior knowledge has led to a greater effect on young adults’ memory than on older adults’ memory (Craik & Masani, 1967; Heron & Craik, 1964). In these studies, meaningless information led to smaller age deficits in memory than meaningful information. As reviewed above, age differences in knowledge use have been studied across a wide variety of memory paradigms. Much prior research in this area has primarily focused on establishing whether older adults *can* show effects of knowledge application, and if so, whether these differ from young adults. At this stage, it is well accepted that prior knowledge can be useful to older adults in memory tasks. Other studies have also demonstrated that older adults have good access to their knowledge: older adults perform better than do young adults in vocabulary tests (Verhaeghen, 2003), show good recall and recognition of general knowledge (Botwinick & Storandt, 1980), and display intact priming (rapid activation of semantic knowledge) (Laver & Burke, 1993). Importantly, what remains to be clarified are the circumstances under which older adults benefit more from knowledge application than young adults and the circumstances under which there is age invariance in the use of knowledge to support memory. Dissociating these effects will elucidate the effects of knowledge on processes related to cognitive aging.

Amongst the studies in the literature that do and do not show disproportionate effects of prior knowledge on older adults’ memory relative to young, there would appear to be no consistent pattern as a variety of different paradigms reveals both types of age effect. Our approach therefore began on an exploratory basis, combining the notions of prior knowledge and environmental support. Linking memory stimuli to prior knowledge has been seen as a method of providing environmental support (Bäckman & Herlitz, 1990; Naveh-Benjamin, Craik, Guez, & Kreuger, 2005); Craik (1986) argued that increasing environmental support reduces cognitive demands and therefore reduces age deficits in memory. It is therefore possible that clarifying age differences in the use of prior knowledge may provide new insight into environmental support and the role of cognitive demands in aging.

Across the first three experiments, we manipulated prior knowledge in line with the environmental support hypothesis, initially on an exploratory basis. This led to the identification of a potentially crucial factor that appeared to determine whether or not older adults’ memory disproportionately benefited from prior knowledge application, which was then tested explicitly in Experiment 4. In brief, prior knowledge was found to be particularly beneficial to older adults’ memory when it provided extra (specific) guidance at retrieval, which operated in addition to the episodic memory itself.

## Experiment 1

This experiment aimed to set a benchmark by maximizing age differences in memory and by maximizing the potential for prior knowledge to aid memory.

The environmental support hypothesis highlights that age deficits in memory are greater when there is less support during retrieval and that they are the greatest during free recall tasks where there are no environmental cues at all at retrieval (Craik, 1986). We therefore presumed that prior knowledge would have the maximum chance of reducing age deficits in memory with a free recall design.

Another feature of this experiment was the use of English and Asian proverbs as memory stimuli (prior knowledge present and absent, respectively). Other studies manipulating prior knowledge have typically used common versus uncommon stimuli but have not always maximized the difference between these conditions. For example, Hess (1985) used actions that were typical or atypical but all of the actions were normal enough to have been encountered in life before the experiment. Arbuckle et al. (1990) tested memory for facts within passages of text about music or dogs (with music experts and nonexperts), but it is not clear whether the participants who were music experts knew the information in one type of passage but not in the other. Our manipulation of prior knowledge was designed to maximize the contrast between known and unknown stimuli. Additionally, we assessed prior knowledge of each proverb after the memory test had taken place.

### Method

#### Design

There were two factors: age (young vs. older adults; between participants) and prior knowledge (present vs. absent; within participants).

#### Participants

Thirty-nine young and 36 older adults took part in the experiment (this excludes one young and two older adults who failed to follow the instructions). Young participants were recruited from the University of Warwick and received either £6 or course credit. Older participants were all living independently and were recruited from an age study volunteer panel populated by local advertisements; they each received £10 toward their travel expenses. All participants were native English speakers.

Background information is summarized in Table 1, where it can be seen that young and older participants did not differ significantly in their years of education, *t* < 1. To assess cognitive functioning, participants completed the Digit Symbol Substitution test from the Wechsler Adult Intelligence Scale–Revised (Wechsler, 1981) as a measure of processing speed, and the multiple choice part of the Mill Hill vocabulary test (Raven, Raven, & Court, 1988) as a measure of crystallized intelligence. The results were consistent with the literature (e.g., Salthouse, 2010) in showing lower speed but higher vocabulary in older than in young adults, *t*(73) = −7.27 and 9.15, respectively.

**Table 1.** Background details for participants in Experiments 1–4.

	Experiment 1		Experiment 2		Experiment 3		Experiment 4	
Variable	Young	Older	Young	Older	Young	Older	Young	Older
N (M/F)^a^	39 (5/34)	36 (8/28)	30 (9/21)	31 (17/14)	29 (13/16)	29 (10/19)	36 (17/19)	36 (17/19)
Age range	18–29	65–86	19–23	65–86	18–30	65–80	18–30	67–81
Mean age (*SD*)	19.5 (1.9)	75.0 (6.9)	20.5 (1.0)	71.4 (5.9)	21.3 (3.0)	71.2 (5.4)	22.7 (2.8)	74.2 (3.8)
Mean years of education (*SD*)	14.3 (1.4)	14.6 (3.9)	16.1 (0.87)	14.7 (3.01)*	12.4 (3.9)	11.8 (2.6)	16.1 (2.5)	16.0 (3.7)
Speed (*SD*)^b^	69.8 (11.9)	50.7 (10.7)**	71.0 (9.6)	41.8 (11.1)**	64.0 (10.9)	41.5 (11.9)**	72.0 (10.9)	50.6 (8.1)**
Vocabulary (*SD*)^c^	16.4 (2.6)	23.0 (3.6)**	18.4 (3.10)	22.8 (4.5)**	17.4 (3.4)	23.2 (3.6)**	17.7 (4.3)	23.2 (4.3)**

Notes: ^a^Number of participants whose data were included in the analyses (males/females).

^b^Mean information processing speed (and standard deviation) based on the Digit Symbol Substitution test (Wechsler, 1981).

^c^Mean vocabulary score (and standard deviation) based on the multiple choice section of the Mill Hill vocabulary test (Raven et al., 1988); maximum score = 33. *Older adults significantly different from young adults, *p* < .05, **p < .001.

#### Materials

The study items were 60 English proverbs and 60 Asian proverbs that were translated into English (see Table 2 for examples). These were all were taken from Poppenk, Kohler, and Moscovitch (2010) who had previously found superior memory for the pre-experimentally familiar English proverbs compared with the novel Asian proverbs. Participants studied 10 English and 10 Asian proverbs for later recall; these were selected randomly for each participant from the full set of materials.

**Table 2.** Examples of stimuli in study and test phases of Experiments 1–4.

Experiment	Study	Test
1	**English and *Asian* proverbs**	**Free recall**
	Don’t put all your eggs in one basket	
	Two wrongs don’t make a right	
	*Talk does not cook rice*	
	*A tree grown in the wind has strong roots*	
2	**Sentences describing scenes**	**Recognition (old/new) of sentences/*pictures***
	High knowledge condition	
	A man sitting on a bench	A man sitting on a bench (old)
	Some toothbrushes by a sink	*Some toothbrushes by a sink* (old)
		A man sitting on a wall (new)
		*Some toothbrushes in a travel bag* (new)
	Low knowledge condition	
	A cat in a tree	A cat in a tree (old)
	Sand on a building site	*Sand on a building site* (old)
		A bird in a tree (new)
		*Sand on a beach* (new)
3	Professional–action pairs*	**Recognition (old/new)**
	The lawyer defended the client in court	The lawyer defended the client in court (old)
	The painter washed all of the brushes	The painter washed all of the brushes (old)
	*The doctor made a sandwich for lunch*	The doctor made a sandwich for lunch (old)
	*The musician drove to the petrol station*	The musician drove to the petrol station (old)
		The lawyer washed all of the brushes (new)
		The painter defended the client in court (new)
		The doctor drove to the petrol station (new)
		The musician made a sandwich for lunch (new)
4	**Word pairs**	**Cued recall**
	Unique relations	
	spear–pistol, horn–trombone, dog–horse…	spear-? horn-? dog-?…
	Shared relations	
	banker–fireman, engineer–cook, athlete–teacher… cherry–pear, orange–apple, plum–lemon…	banker-? engineer-? athlete-?… cherry-? orange-? plum-?…
	No relations	
	whiskey–jacket, hawk–volcano, sycamore–tent…	whiskey-? hawk-? sycamore-?…

Notes: Stimulus order randomized at study (Experiments 1–4) and at test (Experiments 2–4). *Knowledge-consistent pairs in normal type; knowledge-neutral pairs in italics.

#### Procedure

Participants completed five short study-test blocks. In each block, two English and two Asian proverbs were studied for a later free recall test. Proverbs were displayed sequentially at a rate of 7 s per proverb and the order of proverbs was random. Between study and test there was a 20-s delay period where participants responded true/false via a button press as to the correctness of various simple equations (e.g., 4 + 2 = 6; true). At test, participants were instructed to say out loud anything they could remember from the study set in any order they wished. Responses were recorded on a digital Dictaphone for later scoring. The whole study-test procedure was repeated five times, each with a new set of proverbs. In addition, there was a two-proverb practice block at the beginning of the session in order to familiarize participants with the procedure.

After the memory tests were completed, participants were shown all of the proverbs again individually and were asked “Were you familiar with this proverb before the experiment?” to which they responded with keys “J” (yes) and “F” (no).

### Results and discussion

A proverb was scored as accurately recalled if the main gist of the proverb was recalled as well as the majority of words from that proverb (across the whole experiment, in only four cases was the gist recalled without also recalling the majority of words). Performance for each participant was conservatively based only on those English proverbs that he/she reported as knowing prior to the experiment (i.e., high prior knowledge), and those Asian proverbs that he/she reported as not knowing previously (no prior knowledge).^1^ Data were combined across the five study-test blocks. Throughout the article, standard null hypothesis tests are accompanied by an estimated Bayes Factor obtained through JASP computer software (Love et al., 2015). The Bayes Factor (*BF_10_*) provides an odds ratio for the alternative/null hypotheses (values <1 favor the null hypothesis and values >1 favor the alternative hypothesis). For example, a *BF_10_* of 0.40 would indicate that the null hypothesis is 2.5 times more likely than the alternative hypothesis (see Jarosz & Wiley, 2014).

**Figure 1.**
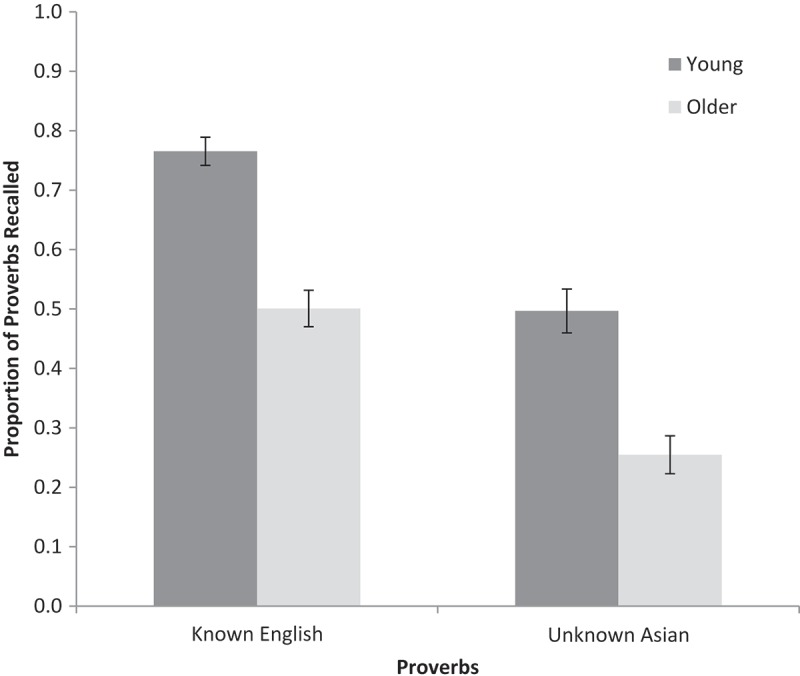
Proportion of proverbs recalled by young and older adults for known (English) and unknown (Asian) proverbs in Experiment 1. Error bars are ±1 *SE*.

A 2 (age: young, older) × 2 (prior knowledge: present, absent) repeated-measures ANOVA was conducted on the proportions of proverbs accurately recalled (see Figure 1 for means). Young adults recalled more than did older adults, *F*(1, 73) = 56.05, *MSE* = 0.04, *p* < .001, *ƞ_p_^2^ *= .43, *BF_10_* = 3.19 × 10^7^. There was a main effect of prior knowledge, *F*(1, 73) = 83.01, *MSE* = 0.03, *p* < .001, *ƞ_p_^2^ *= .53, *BF_10_* = 9.23 × 10^12^, with known English proverbs being remembered better than unknown Asian proverbs. Thus the manipulation of prior knowledge was successful. Crucially, there was no interaction between age and prior knowledge (*F *< 1, *BF_10_* = 0.99). To confirm that the study had sufficient statistical power, power analysis using G*power software (Faul, Erdfelder, Lang, & Buchner, 2007) was used to determine how many participants would be required to achieve a power of .8 to detect a medium effect size (as defined by Murphy & Myors, 1998). To detect a two-way interaction (with one between-participants factor and one within-participants factor) would require 28 young and 28 older participants (i.e., fewer than were tested).

The proverbs in the current study were multicomponential as they contained several words in each memory item. Therefore, the lack of an age by knowledge interaction is partially consistent with the studies presented in the introduction. Of the studies showing age invariance in the effect of prior knowledge on memory, all were based on multicomponent stimuli (e.g., recall for passages of text, Arbuckle et al., 1990; positions of multiple items, Cherry & Jones, 1999). However, some studies showing greater knowledge effects on older adults’ memory relative to young adults’ memory also involved multicomponent information (Garcia-Bajos et al., 2012; Hess, 1985).

Prior knowledge aided memory effectively, but equally so for the two age groups. This indicates that our lack of age variance in prior knowledge use was not due to (1) weak contrast between high and low prior knowledge conditions (which was maximal) or (2) small age-related deficits in memory, which were large (*ƞ_p_^2^ *= .43) due to the free recall design. It has been suggested that prior knowledge effects operate at retrieval (Alba & Hasher, 1983). Therefore, in Experiment 2, we continued to investigate environmental support by manipulating the amount of support available at retrieval, a factor that was crossed with a prior knowledge manipulation. This should help establish whether prior knowledge can provide support beyond that of effective environmental cues.

## Experiment 2

In this experiment, encoding specificity was manipulated by altering the nature of cues provided during retrieval. In line with the encoding specificity hypothesis (Tulving & Thompson, 1973), it was expected that encouraging similar processing at encoding and retrieval would lead to better memory performance. In a novel paradigm, participants studied sentences describing simple scenes and their memory was tested either with the original sentences (high encoding specificity/environmental support) or with pictures of the scenes (low encoding specificity/environmental support). Prior knowledge was used to further influence the match between the sentence and the expected image. For example, for the sentence “A man sitting on a bench”, the image is supported by prior experience and is relatively easy to visualize as there is only one way to sit on a bench and benches are generally quite similar. However, for the sentence “A man sitting on a wall”, the image is much more novel because the man could be sitting across the wall, or leaning against it and the wall could be high or low, etcetera. Therefore, as opposed to Experiment 1 where prior knowledge was either present or absent, here, prior knowledge was either high or low.

Prior research has shown that older adults favor gist-based processing, preferring to remember information in terms of its general meaning as opposed to its surface form (Craik & Simon, 1980; Reder, Wible, & Martin, 1986). This has been hypothesized to limit the specificity of their memory traces, and has been used to explain data showing reduced encoding specificity effects in older adults compared to young adults (Puglisi, Park, Smith, & Dudley, 1988; Rabinowitz, Craik, & Ackerman, 1982). Experiment 2 aimed to manipulate encoding specificity effects by using stimuli that were common or uncommon. Imagery evoked by sentences describing common scenes was expected to match pictures of those scenes more naturally and specifically than sentences and pictures of uncommon scenes. This may therefore reduce the requirement for participants to encode specific memory traces and commensurately may alleviate age-related memory deficits that result from gist-based processing.

### Method

#### Design

There were three factors: age (young vs. older; between participants), prior knowledge (high vs. low; within participants), and encoding specificity (high vs. low; within participants).

#### Participants

Thirty young and 31 older adults took part in the experiment (see Table 1). Young participants were students recruited from the University of Warwick, and independently living older participants were recruited from the local community. Participants were offered no financial incentives for volunteering. Young participants had completed more years of education and produced higher speed scores and lower vocabulary scores than older participants, *t*(33.7) = 2.53, *t*(59) = 11.02, and *t*(59) = −4.50, respectively.

#### Materials

Images were found from the internet and sentences were generated for 30 different pairs of scenes (see Table 2 for example study and test sentences). Within each pair, one was a high prior knowledge scene (something encountered regularly in life or on television; e.g., “Some toothbrushes by a sink”, “A man sitting on a bench”) and the other was a low prior knowledge scene (something encountered less regularly in life or on television; e.g., “Some toothbrushes in a travel bag”, “A man sitting on a wall”). An image was found for each scene and a sentence describing each image was generated. This produced 30 sets of four stimuli, with two images and two sentences.

#### Stimuli validation

Initially, the matched high and low knowledge consistency pairs of stimuli were assessed to establish which would be encountered more often by people. Six independent participants aged 18–50 years (*M* = 26.5, *SD* = 12.2) were presented with the matched pairs of images or pairs of sentences on a sheet of paper and were asked to indicate which item of each pair they see more often in everyday life (including on the television). Each participant saw half of the pairs as images and half of the pairs as sentences. Participants were shown 30 pairs in total and the stimuli chosen for the experiment were those from stimulus sets where at least five out of six participants chose the high prior knowledge scene as more often encountered than the low prior knowledge scene. Twenty-three out of 30 sets met this criterion and 22 were used in the experiment.

In addition, to check that the sentences matched the images, the six participants were given 20 images and 20 sentences to match up. This was repeated three times so that all 60 stimuli (30 high and 30 low knowledge consistency) image-sentence matches were assessed. All six participants scored perfectly on this task, indicating that the images were appropriately described in the sentences.

#### Procedure

The experiment involved encoding sentences and recognizing either sentences (high encoding specificity) or pictures (low encoding specificity). Two separate memory tasks were completed, one with high prior knowledge stimuli at study and one with low prior knowledge stimuli at study, with the order of these blocks counterbalanced across participants.

For an individual block, participants studied 10 sentences sequentially for 5 s each, with a 500-ms interstimulus interval. For each participant, the 22 sets of stimuli were randomly allocated such that 10 were assigned to the high prior knowledge block, 10 to the low prior knowledge block, and two were used for practice. Of the 10 displayed sentences per block, five were randomly chosen to be tested as sentences and five were randomly chosen to be tested as pictures.

Between study and test there was a 30-s delay period where participants responded true/false via a button press as to the correctness of various simple math equations.

For the recognition tests, there were 20 trials with 10 old and 10 new stimuli. The new stimuli were taken from the opposite scene within each stimulus set. For example, if the participant studied “Some toothbrushes by a sink”, then the lure would be “Some toothbrushes in a travel bag” (or the corresponding image). Targets and lures for a given study sentence were always presented in the same format (i.e., both presented as sentences during recognition or both presented as images during recognition). Images were presented in the center of the 1024 × 768 pixel display within a frame of 512 × 384 pixels. The order of recognition trials was entirely random with targets/lures and sentences/images intermixed; stimuli were presented until response, followed by a 500-ms interstimulus interval. The recognition test required participants to press “J” for old/seen-before or “F” for new/not-seen-before with the index fingers of each hand. They were encouraged to respond accurately.

Participants initially completed a practice task in the same format but with just two study sentences and four recognition trials (with materials not used in the main test). After practice, participants completed the two experimental blocks, with a rest period between them.

### Results and discussion

The proportions of hits (the proportion of correct endorsements of old stimuli in the recognition test) and false alarms (the proportion of incorrect endorsements of new stimuli in the recognition test) are presented in Appendix 1. We used the standard corrected recognition measure of performance, scored as the proportion of hits minus the proportion of false alarms.

**Figure 2.**
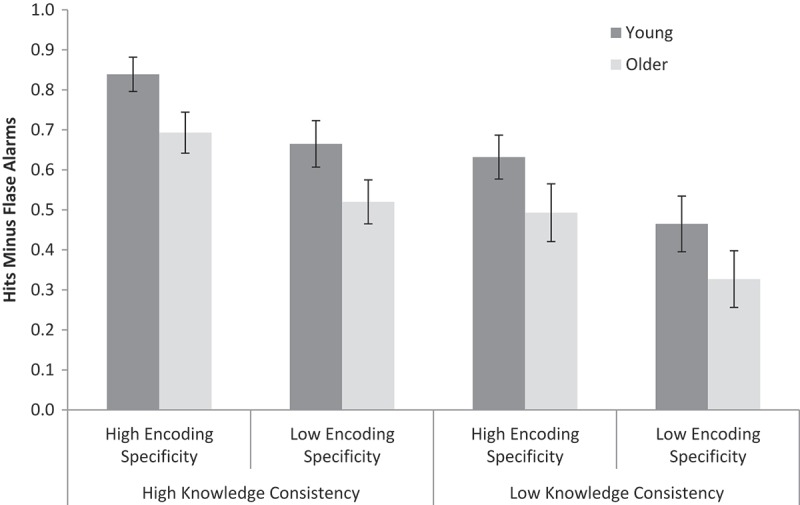
Recognition memory performance (hits minus false alarms) for young and older adults, high and low knowledge consistency stimuli, and high and low encoding specificity recognition trials in Experiment 2. Error bars are ±1 *SE*.

A 2 (age: young, older) × 2 (prior knowledge: high, low) × 2 (encoding specificity: high, low) repeated-measures ANOVA^2^ was conducted on the hits minus false alarms data (see Figure 2 for means). Young adults performed better than older adults, *F*(1, 59) = 6.72, *MSE* = 0.18, *p* < .05, *ƞ_p_^2^ *= .10, *BF_10_ *=* *1.66. High prior knowledge stimuli were recognized better than low prior knowledge stimuli, *F*(1, 59) = 31.97, *MSE* = 0.08, *p* < .001, *ƞ_p_^2^ *= .35, *BF_10_ *= 3.50 × 10^4^. There was also a main effect of encoding specificity, *F*(1, 59) = 19.63, *MSE* = 0.09, *p* < .001, *ƞ_p_^2^ *= .25, *BF_10_ *= 1103, with better memory performance for high than for low encoding specificity recognition trials (i.e., for sentences rather than pictures). None of the interactions was significant (*F*s < 1; Age × Prior knowledge, *BF_10_ *= 0.35; Age × Encoding specificity, *BF_10_ *= 0.32; Age × Prior knowledge × Encoding specificity, *BF_10_ *= 0.02; Prior knowledge × Encoding specificity, *BF_10_ *= 0.43).

Park, Puglisi, Smith, and Dudley (1987) found a congruent result to the current data with young and older adults benefiting similarly from visual cues present at encoding and retrieval to increase encoding specificity. However, surprisingly, prior knowledge did not interact with encoding specificity in the current study despite the strength of both main effects. This indicates that high prior knowledge support did not selectively benefit the low encoding specificity condition compared with the high encoding specificity condition as hypothesized. However, it is still likely that knowledge did help memory by increasing encoding specificity. Visualization can play a large part in memory for verbal material – for example, concrete nouns are easier to remember than abstract nouns (see Paivio, 1991), and mnemonic techniques such as the method of loci and the pegword system are based upon improving memory via visualization techniques (Eysenck, 2009). Given that participants were aware that their memory could later be tested visually and that the study sentences were designed to describe a visual scene, it is highly likely that participants encoded them visually and used these visual representations during retrieval. The verbal to visual translation was by design more specific for the high prior knowledge support stimuli. For example, visualizing a man sitting on a bench will be more specific than visualizing a man sitting on a wall because the details of the bench and how a man might sit on it are more specific than for how a man might sit on a wall.

Crucially, there was absolutely no hint of an age by prior knowledge interaction, or a three-way interaction. Power analysis was conducted as described in Experiment 1. With one between-participants factor and two within-participants factors, the study would require 28 young and 28 older participants to detect an interaction of medium effect size with a power of .8 (again fewer than were tested). These data therefore show that young and older adults make similar use of prior knowledge to encode and retrieve visual descriptions of scenes. The data also suggest that knowledge may facilitate verbal to visual translations of stimuli during encoding and retrieval, again to the same extent in both young and older adults.

## Experiment 3

Experiment 3 continued to focus on how prior knowledge might influence the amount of environmental support available at retrieval, using similar stimuli to Experiment 2, but remaining in the verbal domain. Here we developed a recognition memory test where prior knowledge facilitated the identification of hits *and* the rejection of lures. In old–new recognition tasks, older adults often show particular difficulties in rejecting lures. For example, Reder et al. (1986) showed that after memorizing short stories, older adults were more likely to endorse lure sentences than young adults when those lures were plausible. Cohn, Emrich, and Moscovitch (2008) found that after studying word pairs, older adults performed similarly to young adults in correctly recognizing intact pairs but older adults produced more false alarms than young adults when rejecting rearranged pairs. Older adults have also been shown to be more confident than young adults when incorrectly endorsing lures (e.g., McCabe, Roediger, McDaniel, & Balota, 2009; Shing, Werkle-Bergner, Li, & Lindenberger, 2009).

Participants studied person–action sentences that were either consistent with prior knowledge (e.g., “The teacher marked the mock exam papers”, “The pilot landed the aeroplane safely”) or neutral in relation to prior knowledge (e.g., “The teacher caught the train to London”, “The pilot bought a new winter coat”). When two consistent sentences were rearranged to form recognition lures, they became inconsistent with prior knowledge (e.g., “The teacher landed the aeroplane safely”), but when two neutral sentences were rearranged to form recognition lures they remained neutral (e.g., “The teacher bought a new winter coat”). This design created lures that were relatively easy to reject in the high prior knowledge consistency condition, which was hypothesized to disproportionately favor older adults who have a greater tendency than young adults to endorse lures during recognition.

This type of stimulus is also used in studies of the fan effect (Anderson, 1974) and fan size was manipulated in the current experiment. We have summarized the fan effect data in Appendix 2 as it is not relevant to the current narrative (the fan effect was marginal and did not interact with age or prior knowledge factors). The data presented here do not include the factor relevant to the fan effect.

### Method

#### Design

There were two factors: age (young vs. older; between participants) and prior knowledge (consistent vs. neutral with respect to prior knowledge; within participants).

#### Participants

Twenty-nine young and 29 older adults took part in the experiment for no financial reward (this excludes one young adult who failed to follow the instructions and one older adult whose data were corrupted during the experimental run). Young participants were recruited from the University of Warwick and from the local community, and independently living older participants were recruited from the local community (see Table 1 for details). Young and older participants did not differ in their years of education, *t *< 1, but again there were significantly higher speed scores, *t*(56) = 7.50, and lower vocabulary scores, *t*(56) = −6.25, for young compared with older adults.

#### Materials

Participants studied 16 sentences and then completed 32 old/new recognition trials. This procedure was repeated five times in total with exactly the same stimuli in randomized order, allowing participants to learn the sentences better upon each repetition. Sentences comprised a professional person followed by an action. Study and test sentences were created from 24 actions that were linked to eight professional people consistently with prior knowledge. A further 16 actions were neutral with respect to all professionals (see Table 2 for examples).

Each participant studied eight sentences where the professional and action were consistent with prior knowledge and eight sentences where the professionals were paired with neutral actions. Eight different professionals were used with 16 different actions: four of the professionals were associated with three actions and four were associated with just one action (providing a design suitable for assessing the fan effect – see Appendix 2). Within any given study list, each professional either appeared with only knowledge-consistent actions or only knowledge-neutral actions.

For the recognition test, the original 16 sentences made up half of the trials for the old/intact trials. The remaining 16 lure sentences were created using the same professionals and actions and these were rearranged to form new/recombined trials. The sentences were rearranged within their prior knowledge consistency condition: professional–action pairings consistent with prior knowledge were recombined to form lures *inconsistent* with prior knowledge (e.g., “The teacher landed the aeroplane safely”). In the case of the neutral stimuli, rearranged professional–action pairings remained neutral. This method of recombination ensured that lures were constructed from sentences of the same prior knowledge condition so that they could be distinguished from each other during analysis of recognition trials.

Professionals and actions were combined and recombined entirely randomly (in line with the constraints outlined above) for each participant. All study trials and test trials were presented in a randomized order and this order was randomized anew upon each of the five study-test periods.

#### Procedure

During encoding, participants studied the sentences sequentially at a rate of 5 s per sentence with a 500-ms interval between sentences. They were instructed to remember which professionals were associated with which actions for a later recognition test. Participants were then immediately asked via an on-screen prompt to “Press the space bar to begin the memory test”. They were required to press the “J” key for old/seen-before sentences and the “F” key for new/recombined sentences with the index fingers of each hand and they were instructed to respond as accurately and as quickly as possible. At the end of each study-test period, participants were instructed to “Press space to repeat [the] study list”. Stimuli were presented in a white font on a black background with a letter height corresponding to approximately 1° viewing angle.

### Results and discussion

**Figure 3.**
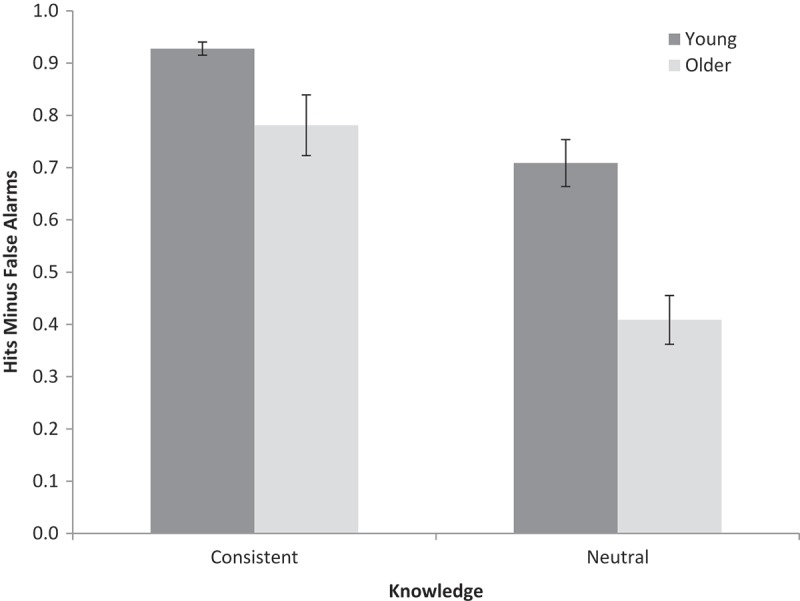
Mean recognition performance (hits minus false alarms) for stimuli consistent or neutral with respect to prior knowledge in Experiment 3. Error bars are ±1 *SE*.

Recognition accuracy was calculated as hit rates minus false alarm rates across all five test runs combined (Appendix 1 shows hits and false alarms separately; Appendix 2 shows the same analysis with test run and fan effect entered as factors). A 2 (age: young, older) × 2 (prior knowledge: consistent, neutral) repeated-measures ANOVA was conducted on accuracy (see Figure 3 for means). Young adults performed better than older adults, *F*(1, 56) = 17.06, *MSE* = 0.09, *p* < .001, *ƞ_p_^2^ *= .23, *BF_10_* = 547. Memory was better for knowledge-consistent phrases than for knowledge-neutral phrases, *F*(1, 56) = 93.34, *MSE* = 0.28, *p* < .001, *ƞ_p_^2^ *= .63, *BF_10_* = 7.92 × 10^10^, indicating that knowledge was supporting memory. Crucially, in contrast to Experiments 1 and 2, there was an age by knowledge consistency interaction, *F*(1, 56) = 6.28, *MSE* = 0.03, *p* < .05, *ƞ_p_^2^ *= .10, *BF_10_* = 13.0, with older adults showing a greater benefit from prior knowledge use relative to young adults (Appendix 2 also shows the same interaction for the first test run only to ensure that the result is not due to ceiling effects in young adults).^3^

Why was the age by knowledge interaction significant here but not for Experiments 1 and 2? Experiments 1 and 2 used a prior knowledge manipulation that was based on frequency of occurrence. In Experiment 2, the difference between high and low prior knowledge stimuli (e.g., “A man sitting on a bench” and “A man sitting on a wall”, respectively) was that the former had been encountered more frequently in everyday life. The same can be said of Experiment 1, although the contrast was greater in that prior knowledge was either present or absent. Experiment 3 differed because it used prior knowledge to construct logical or illogical stimuli (e.g., “The teacher marked the mock exam papers” or “The teacher landed the aeroplane safely”, respectively). Thus prior knowledge supported memory along a separate dimension, providing an *extra* criterion upon which to accept and reject recognition test stimuli that was independent of the memory trace itself. This hypothesis is tested below.

## Experiment 4

It can be seen in the literature that many of the memory tests where prior knowledge disproportionately benefited older adults involved knowledge that was uniquely applicable to each memory that was tested (Badham et al., 2012; Naveh-Benjamin et al., 2003). For example, in Badham et al.’s prior knowledge condition, participants memorized associations between words that were related – participants studied 15 word pairs that were related in different ways (e.g., motorcycle–car = modes of transport, fox–dog = animals, etc. in the same memory list). In these studies, prior knowledge provided extra cueing information that could be used during retrieval, namely relations based on prior knowledge: for example, if a participant was provided with the cue “motorcycle” at test, they knew that the target was (1) shown with that cue earlier, *and* (2) related to that cue.

In contrast, many of the studies that showed similar knowledge effects on memory with young and older adults used conditions that drew upon the degree of exposure to prior knowledge (e.g., music experience, Arbuckle et al., 1990; and cooking experience, Miller, 2003). Here, knowledge helped memory by linking stimuli to wider experience (possibly by encouraging deeper processing, cf. Craik & Lockhart, 1972), but it did not provide extra information, beyond that stored in the memory traces themselves, that could be used to distinguish between memories in the face of interference.

In this final experiment, we manipulated whether or not prior knowledge could be used as an additional cue to guide retrieval. Using a cued recall paradigm similar to Badham et al. (2012), we tested participants’ memory for related and unrelated word pairs. Crucially, two conditions were created with related pairs: unique relations (where every one of 16 word pairs within a list was related in a different way) and shared relations (where multiple word pairs within a list were related in the same way, e.g., eight pairs of transport words plus eight pairs of animal words). In this latter condition, relations were present but provided no additional information to narrow down retrieval options. For example, after studying eight pairs of transport words, remembering that a target word was related to a cue “motorcycle” would not aid in distinguishing it from the seven other transport-based target words. It was hypothesized that older adults would disproportionately benefit from unique relations but not from shared relations.

### Method

#### Design

There were two factors: age (young vs. older; between participants) and word relations (unique relations, shared relations, no relations; within participants).

#### Participants

Thirty-six young and 36 older adults took part in the experiment (see Table 1). Young participants were students recruited from the University of Warwick; older adults were all living independently and were recruited from an age study volunteer panel populated by local advertisements. Participants were compensated £6 for volunteering. Young and older adults had completed similar years of education, *t *< 1. Young participants produced higher speed scores and lower vocabulary scores than older participants, *t*(64.5) = 9.46, *t*(70) = −5.44, respectively.

#### Materials

Groups of words were taken from Van Overschelde, Rawson, and Dunlosky (2004), where participants were required to list as many items as they could that fitted into various categories (e.g., *a fish, a unit of distance*). Categories that contained items from a variety of contexts were excluded (e.g., *a thing made of wood, a thing taken from a burning home*). Also categories containing proper nouns were excluded (e.g., *a country, a football team name*). From their study, 35 categories were used (see Table 3), each containing between 7 and 16 items (where categories contained more than 16 items, the top 16 were taken).

**Table 3.** Categories used in Experiment 4.

Category
A bird
A carpenter’s tool
A chemical element
A fish
A four-footed animal
A fruit
A gardener’s tool
A kitchen utensil
A member of the clergy
A metal
A musical instrument
A natural earth formation
A nonalcoholic beverage
A part of a building
A part of the human body
A precious stone
A relative
A sport
A substance for flavoring food
A transport vehicle
A tree
A type of fabric
A type of footwear
A type of human dwelling
A type of reading material
A unit of distance
A unit of time
A vegetable
A weapon
A weather phenomenon
An alcoholic beverage
An article of clothing
An article of furniture
An insect
An occupation or profession

Categories with 16 items were selected to form the “shared relations” condition. This allowed eight pairs of related words to be formed where the relation was of the same nature for all eight pairs (words within a category being paired together randomly). Two 16-item/8-pair categories were used in this way to form a list of 16 word pairs. The two categories were chosen such that they did not have similar items (e.g., *a fruit* and *a vegetable* would not be combined).

From the remaining categories, 16 different categories were used to form the “unique relations” condition. Two words from each category were randomly chosen to form 16 separate related-items word pairs. Again we ensured that no categories with similar items appeared in the same list.

Finally, 32 categories were used to form the “no relations” condition. Sixteen word pairs were formed with the constraints that no two words of a pair were from the same category and also that spurious relations between words within pairs were avoided whenever possible (see Table 2 for example pairings).

Across all three lists, no items appeared twice for a given participant. Six versions of the experiment were created for counterbalancing (six sets of three study lists). Categories were selected at random in all six versions under the constraint that across the six versions, no category was used more than once in the “shared relations” condition. Across all the lists generated, alliteration and rhymes within pairs were avoided and the mean number of letters per word was six for both the left and right words of each pair. The English Lexicon Project (Balota et al., 2007) was used to assess the frequency of use in language for the words in each condition based on log SUBTL frequency (Brysbaert & New, 2009). The words in each condition occurred with similar frequencies (*F* < 1; unrelated: *M* = 2.59, *SD* = 0.71; unique relations: *M* = 2.61, *SD* = 0.80; shared relations: *M* = 2.51, *SD* = 0.70). The order of pair presentation within lists was randomized for each participant every time the experiment was run.

#### Procedure

Participants were presented with the word pairs sequentially and were asked to remember the associations between the words of each pair. They were informed that they would later be shown the left word of each pair and would be asked to verbally recall the word originally presented alongside it. In order to prevent ceiling effects in young adults and floor effects in older adults, young and older participants received the words at different presentation rates (3 s and 6 s per pair, respectively) as is typical in these types of cued recall memory tests (e.g., Naveh-Benjamin, 2000). There was a 500-ms blank screen between each pair. Between study and test, participants completed 30 s of true/false math questions as described in Experiment 1. For cued recall, participants were shown the left word of each pair and were asked to verbally report the word that it was originally paired with whilst their voice was digitally recorded. Participants were given as long as they needed to recall each word and the experimenter pressed a button once a response was made to present the next cue word. There was a 500-ms interstimulus interval between each word. The cue words were selected in a random order. Participants completed the same study-test procedure three times, once with each type of relation condition. They also completed a short practice test first with two related and two unrelated word pairs. The six possible orders of conditions were crossed with the six sets of experimental stimuli to produce 36 versions of the experiment (one for each young and older participant).

### Results and discussion

**Figure 4.**
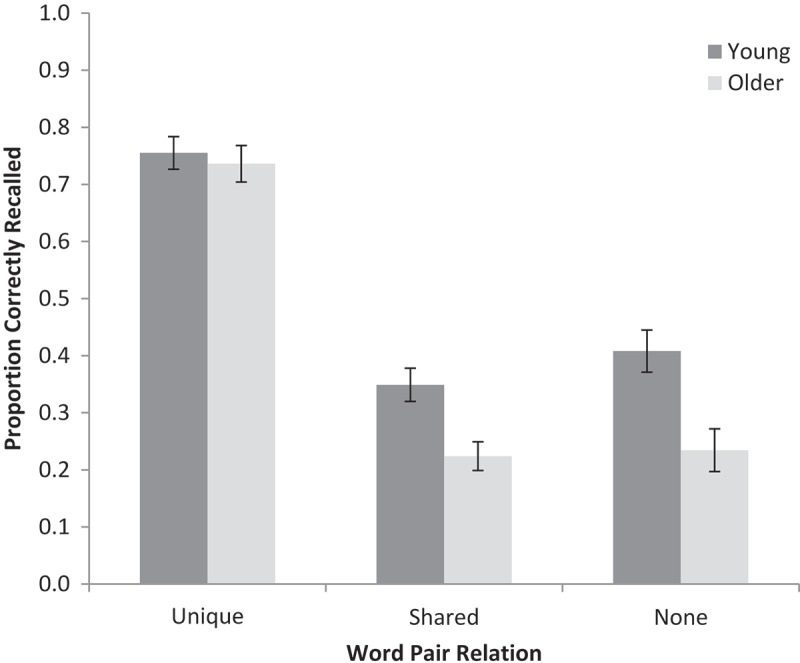
Mean proportion correct for cued recall of word pairs with unique, shared, or no relations. Error bars are ±1 *SE*.

A 2 (age: young, older) × 2 (word relations: unique, shared, none) repeated-measures ANOVA was conducted on the proportion of correctly recalled words (see Figure 4 for means).^4^ Young adults recalled more words than did older adults, *F*(1, 70) = 8.42, *MSE* = 0.07, *p* < .001, *ƞ_p_^2^ *= .11, *BF_10_* = 59.8. There was a main effect of word relations, *F*(2, 140) = 249.88, *MSE* = 0.02, *p* < .001, *ƞ_p_^2^ *= .78, *BF_10_* > 10^12^, and this interacted with age, *F*(2, 140) = 5.97, *MSE* = 0.02, *p* < .01, *ƞ_p_^2^ *= .08, *BF_10_* = 36.1. Planed 2 × 2 comparisons revealed that older adults disproportionately benefited from unique relations relative to no relations compared to young adults, *F*(1, 70) = 12.78, *MSE* = 0.02, *p* < .001, *ƞ_p_^2^ *= .16, *BF_10_* = 102.3, replicating the existing literature (e.g., Badham et al., 2012; Naveh-Benjamin et al., 2003). Crucially, with respect to the current hypothesis, older adults also disproportionately benefited from unique relations relative to shared relations compared to young adults, *F*(1, 70) = 5.79, *MSE* = 0.02, *p* < .05, *ƞ_p_^2^ *= .08, *BF_10_* = 6.23. This demonstrates that relations were only disproportionately helpful to older adults when use of those relations pointed specifically to a target item in memory (i.e., when relations provided an extra strategy by which to identify target words). Furthermore, an age by word relation interaction was absent for the analysis comparing just the shared relation and no relation pairs, *F* < 1, *BF_10_* = 0.43 (words from the shared relations condition being no better recalled than words from the no relations condition, *F* < 1.94, *BF_10_* = 0.39), indicating that relations were not disproportionately helpful to older adults if those relations were not useful for guiding retrieval. The data therefore support our hypothesis and suggest that relations are only disproportionately beneficial to older adults relative to young adults if they provide extra information to guide retrieval extrinsically to the memory trace itself.

## General discussion

In line with existing literature, all of the experiments reported showed that both young and older adults could make use of prior knowledge to improve their memory performance. In Experiment 1, participants memorized proverbs that they had either experienced before or not. This maximized the use of prior knowledge by having it either present or absent. Despite significant age deficits in memory and effects of prior knowledge, the two age groups utilized prior knowledge to the same extent. In Experiment 2, old–new recognition memory for more-common scenes was superior to memory for less-common scenes, again equally for young and older adults (and increasing encoding specificity benefited both age groups similarly).

Crucially, the series of experiments indicated a factor that could explain why older adults sometimes benefit more than young adults when prior knowledge can be applied in memory tasks. Experiment 3 used a high prior knowledge condition with an old/new recognition task where targets were logical and lures were illogical with respect to prior knowledge (e.g., “The teacher marked the mock exam papers”, “The teacher landed the aeroplane safely”, respectively). Here, prior knowledge disproportionately benefited older adults relative to young adults and we hypothesized that prior knowledge operated as an additional cue in the memory task: The concept “is the phrase consistent with my knowledge of the world?” could be used *in addition to memory* to endorse targets and reject lures. This hypothesis was tested in Experiment 4. Young and older participants studied pairs of words that were related (e.g., flood–rain) or unrelated (e.g., crow–sock) and had to later recall the right word of each pair when given the left. Therefore, at test, prior knowledge could be utilized by the concept “the target was related to the cue” in addition to the memory trace. When this concept was useful, older adults disproportionately benefited from relations compared to no relations relative to young adults. However, we constructed an additional condition where the concept “the target was related to the cue” was useless because multiple words were related in the same way (e.g., many weather-related word pairs: flood–rain, snow–tornado, typhoon–sleet, etc.). Here, the concept “the target was related to the cue” was useless because many targets were related to the cue. In this condition, young and older adults showed similar effects of prior knowledge where it could not be used in addition to memory.

The current study therefore suggests that prior knowledge is helpful to older adults in memory tasks, but that it is particularly helpful when it can be used strategically to access appropriate episodic memories. Studies show reduced strategic processing in older adults during memory tasks (Craik & Byrd, 1982; Dunlosky & Hertzog, 2001) and that age-related memory deficits are alleviated when strategic processing is encouraged/facilitated (Glisky, Rubin, & Davidson, 2001; Naveh-Benjamin, Brav, & Levy, 2007). It is therefore evident that prior knowledge may help memory in two dissociable ways: (1) by enhancing the encoding/storage/retrieval of the episodic memory itself, which is an age-invariant process; and (2) by providing extra strategic support (akin to environmental support, Craik, 1986), in addition to the episode itself, which is disproportionately beneficial to older adults relative to young adults.

The current hypothesis can be used to evaluate the contrasting studies in the introduction where there was both age variance and invariance in the use of prior knowledge in memory tasks. First, for studies showing greater prior knowledge effects in older adults compared to young: Smith et al. (1998), Naveh-Benjamin et al. (2003), and Badham et al. (2012) tested associative memory, with prior knowledge providing unique relations between each pair similar to the unique relations condition of the current Experiment 4. Therefore the concept “the items were related” facilitated memory independently of the episodic memory itself. Castel (2005) tested memory for item–price associations that were either realistic or unrealistic – therefore the concept “the price could be plausible” facilitated memory for the realistic prices in addition to the episodic memories. Hess (1985) and Garcia-Bajos et al. (2012) tested young and older adults’ memory for typical and atypical actions performed by characters in events. Therefore the concept “what would someone normally do in this situation?” facilitated memory for typical actions independently of the memory trace itself.

For the studies showing similar effects of prior knowledge for young and older adults, Arbuckle et al. (1990) and Miller (2003) tested young and older experts on information related or not related to their domain of expertise (music and cooking). Here, prior knowledge (expertise) was only useful for enriching memory and the two age groups benefited similarly. The prior knowledge concept “it was related to my music expertise” would not facilitate memory for a whole passage about music. This is similar to the shared relations condition of the current Experiment 4 (where participants studied, for instance, a whole set of word pairs related to weather phenomena). Morrow et al. (1998) assessed memory for appointments in young and older adults where the information was presented in an ordered (knowledge-consistent) or disordered (knowledge-inconsistent) sequence. The concept “the information was ordered logically” would not provide an additional cue to access the information itself. Similarly, Cherry and Jones (1999) tested young and older adults’ memory for spatial locations of furniture that was either organized or unorganized (e.g., all the kitchen furniture together or all the kitchen furniture dispersed randomly). The concept “all the kitchen furniture was together” would not necessarily facilitate memory for the spatial positions of individual items. Although it could be argued that furniture being organized into realistic positions would provide an external cue via the concept “the furniture should be logically arranged”, at least one other study has found greater organizational benefits with furniture positions for older adults compared to young adults (Hess & Slaughter, 1990), but note that there are many ways to arrange furniture so logic may not always be helpful. Finally, Gutchess and Park (2009) marks an exception to our hypothesis: groups of young and older participants looked at a series of images with a central picture (e.g., a cow) presented in front of a regular (e.g., farm) or irregular (e.g., laundry room) background. We would predict that the concept “the picture fits with the background” would particularly help older adults retrieve regular picture–background associations. This design is similar to the uniquely related word pairs in our Experiment 4, but Gutchess and Park found that young and older adults benefited from regularity to the same extent. However, there were few effects of relatedness across the three experiments in Gutchess and Park (2009), which might have limited their chance of finding an age by relatedness interaction.

One methodical issue that deserves some consideration here is the influence of scaling effects, whereby overall differences in performance between young and older adults may lead to the presence of spurious interactions, or indeed the absence of real interactions (Salthouse, 2000). Throughout the article we have been careful to avoid ceiling effects in young adults’ memory and floor effects in older adults’ memory that can drive interactions. Other research such as Naveh-Benjamin et al. (2003) has also shown that prior knowledge can disproportionately benefit older adults compared to both a full attention young group, who performed better than the older adults, and a divided attention (at encoding) young group, who performed equivalently to older adults overall. Additionally, the main effect of age varied in magnitude between Experiments 3 and 4 (*ƞ_p_^2^ *= .23 and *ƞ_p_^2^ *= .11, respectively), both of which showed age by prior knowledge interactions, and between Experiments 1 and 2 (*ƞ_p_^2^ *= .43 and *ƞ_p_^2^ *= .10, respectively), both of which showed no age by prior knowledge interactions. This demonstrates that a variety of overall age differences can yield both the presence and absence of age differences in the use of prior knowledge. Thus we observed no consistent influence of scaling effects on age differences in the use of prior knowledge in the current data.

To summarize, these results and much of the existing literature support our hypothesis that prior knowledge effects on memory can be dissociated into two types. First, on an age-invariant basis, prior knowledge improves memory traces themselves. A possible mechanism for this is the levels of processing framework (Craik & Lockhart, 1972), where prior knowledge can be seen to encourage deeper processing of stimuli, resulting in more effective encoding. Arbuckle et al. (1990) argued that prior knowledge may also aid in organizing and structuring concepts in memory to produce more relevant chunks (cf. Miller, 1956). And many notions from schema theory might be relevant to this effect (Alba & Hasher, 1983). Second, prior knowledge may act to support memory, independently of the memory trace itself. This can be achieved by providing participants with information that can be used in addition to memory, about the structure of a given memory task or memory stimuli. For example, being aware that a target is semantically related (linked by prior knowledge) to a cue can aid in accessing that target (Experiment 4), and this awareness requires no access to the memory trace itself. This property of prior knowledge is particularly beneficial to older adults’ memory and we have demonstrated here that it can alleviate age-related deficits in associative memory.

Finally, there are other aspects to the use of prior knowledge that have not been covered in the current article. Information incongruent with prior knowledge can be difficult to remember, often particularly so for older adults relative to young (see Umanath & Marsh, 2014, for a review). Other complications arise when the linking of experimental stimuli to prior knowledge is effortful, which can cause reductions in memory performance, particularly for older adults (Badham & Maylor, 2015). Overall, there is much literature regarding the use of knowledge by older adults and it remains an important issue to elucidate age variance in its access and application in cognition.

## Figures and Tables

**Figure A1.**
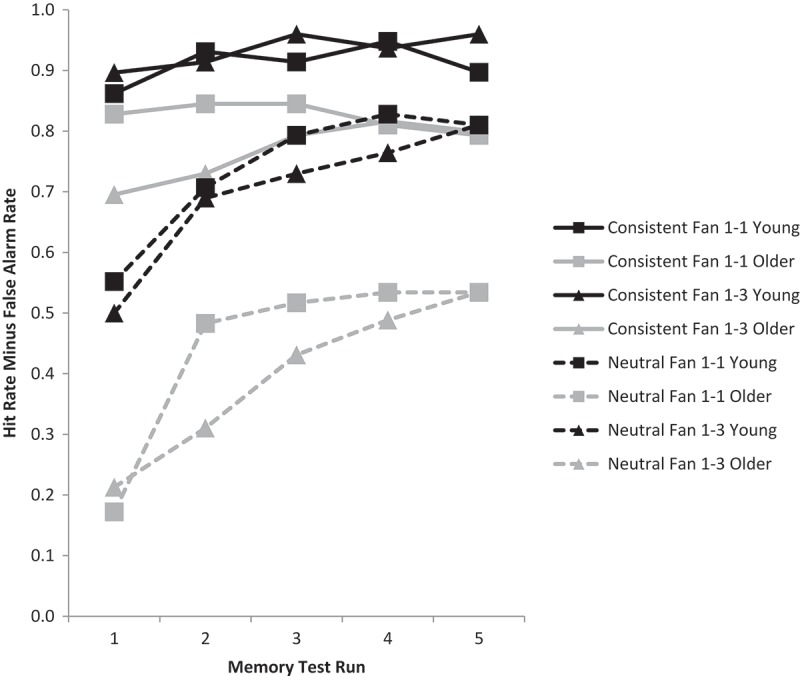
Mean recognition performance (hits minus false alarms) across five test runs for young and older adults, knowledge-consistent and knowledge-neutral stimuli, and 1–1 and 1–3 fan levels in Experiment 3.

## Notes

1. Young adults reported knowing significantly fewer English proverbs (out of 10) than did older adults: *M*_young_ = 8.6, *SD*_young_ = 1.2; *M*_older_ = 9.7, *SD*_older_ = 0.6; *t*(58.90) = 5.07, *p* < .001. However, young and older adults reported not knowing similar numbers of Asian proverbs (out of 10): *M*_young_ = 9.1, *SD*_young_ = 1.3; *M*_older_ = 9.4, *SD*_older_ = 1.3; *t*(73) = 1.00, *p* = .32. The pattern of results in the main ANOVA for Experiment 1 was identical when including all English proverbs in the prior knowledge condition and all Asian proverbs in the no prior knowledge condition.

2. Separate analysis confirmed that there was no significant influence of test order and no interactions between test order and any of these factors.

3. In addition, for response times to hits, there were highly significant effects of age, knowledge consistency, and age × knowledge consistency, all *p*’s < .001, such that older adults showed much greater speeding up from the correct recognition of knowledge-neutral (2445 ms) to knowledge-consistent (1832 ms) phrases than did young adults (from 1351 to 1278 ms).

4. An initial three-way ANOVA showed no significant main effect of condition order and no interactions involving condition order.

## Appendix 1.

**Table A1.** Means (and standard deviations) for hit and false alarm rates in young and older adults in Experiments 2 and 3.

	Young		Older	
	Hits	False alarms	Hits	False alarms
**Experiment 2**				
High knowledge consistency – High encoding specificity	.884 (.153)	.045 (.123)	.873 (.162)	.180 (.219)
High knowledge consistency – Low encoding specificity	.800 (.193)	.135 (.189)	.753 (.261)	.233 (.204)
Low knowledge consistency – High encoding specificity	.839 (.209)	.206 (.203)	.807 (.270)	.313 (.296)
Low knowledge consistency – Low encoding specificity	.723 (.229)	.258 (.214)	.700 (.286)	.373 (.256)
**Experiment 3**				
Knowledge-consistent	.968 (.039)	.041 (.041)	.916 (.149)	.135 (.226)
Knowledge-neutral	.885 (.094)	.177 (.173)	.825 (.113)	.416 (.214)

## Appendix 2. Measurement of the fan effect in Experiment 3

During encoding, participants were shown eight professionals associated with 16 actions (eight actions for knowledge-consistent stimuli and eight actions for knowledge-inconsistent stimuli). Some professionals were associated with three actions (providing a fan level of 1–3) and some were associated with just one action (providing a fan level of 1–1). The fan effect hypothesis states that accuracy is poorer and response times slower when a single item is associated with multiple other items. This is due to cue overload where items interfere with each other during retrieval as multiple items are activated by a single cue. Older adults are more affected by fan size than are young adults, showing disproportionately more recognition errors for larger fan sizes compared to smaller fan sizes (Gerard, Zacks, Hasher, & Radvansky, 1991; Radvansky, Zacks, & Hasher, 1996) and disproportionately slower responses for larger fan sizes compared with smaller fan sizes (Cohen, 1990; Gerard et al., 1991).

A 2 (age: young, older) x 2 (knowledge consistency: consistent, neutral) x 2 (fan level: 1–1, 1–3) x 5 (test run: one to five) repeated-measures ANOVA was conducted on accuracy (hits minus false alarms; see Figure A1 for means). Young adults performed better than older adults, *F*(1, 56) = 14.92, *MSE* = 0.87, *p* < .001, *ƞ_p_^2^ *= .21. There was a main effect of knowledge consistency, *F*(1, 56) = 85.24, *MSE* = 0.28, *p* < .001, *ƞ_p_^2^ *= .60, with better memory for knowledge-consistent phrases than for knowledge-neutral phrases. The effect of fan level did not reach significance, *F*(1, 56) = 2.30, *MSE* = 0.13, *p* = .14, *ƞ_p_^2^ *= .04, although the means were in the predicted direction of superior performance for fan level 1–1 than for fan level 1–3. There was also a main effect of test run, *F*(3.22, 180.55) = 16.43, *MSE* = 0.10, *p* < .001, *ƞ_p_^2^ *= .23, with performance improving across test runs as participants learned the stimuli. There was an age by knowledge consistency interaction, *F*(1, 56) = 7.41, *MSE* = 0.28, *p* < .01, *ƞ_p_^2^ *= .12, with older adults showing a greater benefit from knowledge consistency relative to young adults. There was also an interaction between test run and knowledge consistency, *F*(3.33, 186.52) = 9.59, *MSE* = 0.08, *p* < .001, *ƞ_p_^2^ *= .14, with performance for knowledge-neutral sentences benefiting more across test runs than knowledge-consistent sentences. There were no other interactions.

In addition, to address the possibility that the age x knowledge consistency interaction was driven by ceiling effects in young adults, the same repeated-measures ANOVA was conducted on the hit minus false alarm rates data for the first test run only. The effects were all as before, importantly including the age by knowledge consistency interaction, *F*(1, 56) = 4.61, *MSE* = 0.15, *p* < .05, *ƞ_p_^2^ *= .08.
